# Can Lipoprotein-associated Phospholipase A2 be Used as a Predictor of Long-term Outcome in Patients with Acute Coronary Syndrome?

**DOI:** 10.2174/1573403X09666131202143349

**Published:** 2013-11

**Authors:** Sine Holst-Albrechtsen, Maria Kjaergaard, Anh-Nhi Thi Huynh, Johanne Kragh Sorensen, Susanne Hosbond, Mads Nybo

**Affiliations:** 1 Dept. of Clinical Biochemistry and Pharmacology; 2 Dept. of Cardiology, Odense University Hospital, Denmark

**Keywords:** Acute coronary syndrome, biomarker, Lp-PLA2, mortality, plaque rupture, prospective studies, statins.

## Abstract

Studies indicate that elevated plasma concentrations of lipoprotein-associated phospholipase A2 (Lp-PLA2) is associated with increased risk of cardiovascular disease. Lp-PLA2 seems to play a crucial role in the formation of plaques and acute inflammation, and plasma Lp-PLA2 could therefore potentially be used as a predictor of long-term outcome in ACS patients. To evaluate this, data concerning Lp-PLA2 as a predictor in ACS patients was gathered through a systematic literature review, and studies on this issue were extracted from relevant databases, incl. PubMed and Cochrane. A total of 14 articles were retrieved, but after thorough evaluation and elimination of irrelevant articles only seven studies were eligible for the literature review. All studies except two showed significant correlation between Lp-PLA2 and CV events in ACS patients. Only one study found an independent value to predict CV events 30 days after ACS. Altogether, there was inconsistency in the findings regarding the potential use of Lp-PLA2 and a lack of knowledge on several issues. Lp-PLA2 seems to give valuable information on which ACS patients are prone to new events and also provides important information on plaque size. However, more focused studies concerning genetic variations, time-window impact, patients with and without CV risk factors (e.g. diabetes), and treatment effects are needed. In conclusion, Lp-PLA2 offers new insight in the pathophysiological development of ACS, but until the aforementioned issues are addressed the biomarker will mainly be of interest in a research setting, not as a predictive parameter in a clinical setting.

## INTRODUCTION

Today, the diagnostic criteria for myocardial infarction (MI) is detection of a rise or fall of cardiac biomarkers (especially troponins) and one of the following: symptoms of ischemia, electrocardiography (ECG) changes, development of pathological Q-waves in the ECG, and imaging evidence of new loss of viable myocardium or new regional wall motion abnormality. Further individual diagnostic criteria for MI are sudden unexpected cardiac death, percutaneous coronary intervention (PCI) patients, coronary artery bypass graft (CABG) patients and pathological findings of MI [1].

Cardiac troponins as an early diagnostic biomarker has major relevance in the diagnosis of MI, but is only available in a limited time window and does only provide limited information for some patient categories[2]. Several other biomarkers, e.g. C-reactive protein (CRP) and interleukin 6 (IL-6), have been identified also to indicate acute coronary syndrome (ACS) [3]. However, in order to identify the patients at risk and prevent development of ACS, it is important to have a biomarker that indicates the presence of a thin fibrous cap and rupture-prone plaques [3]. A good candidate for this is lipoprotein-associated phospholipase A2 (Lp-PLA_2_), also known as platelet activating factor-acetylhydrolase (PAF-AH). It is a member of the phospholipase A_2_ superfamily and is secreted by macrophages and foam cells in atherosclerotic plaques [4, 5]. In humans, approximately 80% of Lp-PLA_2_ is bound to apolipoprotein B in circulating low density lipoprotein (LDL) cholesterol particles [6]. The oxidation of LDL to oxLDL provides oxidized phospholipids, which is hydrolyzed by Lp-PLA_2_. This produces lysophosphatidylcholine and non-esterified fatty acids, important mediators of inflammation that up-regulates the expression of adhesion molecules activating leukocytes and recruiting macrophages and monocytes to atherosclerotic plaques [4]. Importantly, Lp-PLA_2_ has been found strongly expressed in the vicinity of macrophages of vulnerable and ruptured plaques [3]. Lp-PLA_2_ therefore seems to play a crucial role in the formation of plaques and acute inflammation.

Studies have shown that elevated plasma Lp-PLA_2_ levels are associated with increased risk of cardiovascular disease (CVD) in a healthy population as well as in patients with vascular disease [4]. The West of Scotland Coronary Prevention Study (WOSCOPS) was the first study that showed an association between Lp-PLA_2_ and CVD risk. Hereafter, several other epidemiological studies have found the same association between elevated plasma Lp-PLA_2_ and the development of coronary heart disease (CHD) or CVD [5, 7]. Recently, studies on plasma Lp-PLA_2_ as a possible predictor of long-term outcome in ACS patients have emerged, but the evidence has not yet been evaluated in a structured fashion [3]. The aim of this systematic literature review was to assess, whether plasma Lp-PLA_2_ can be used as a biomarker to predict outcome in ACS patients in order to optimize medical care in this vulnerable patient category.

## MATERIALS AND METHODS

**Fig. (1):**
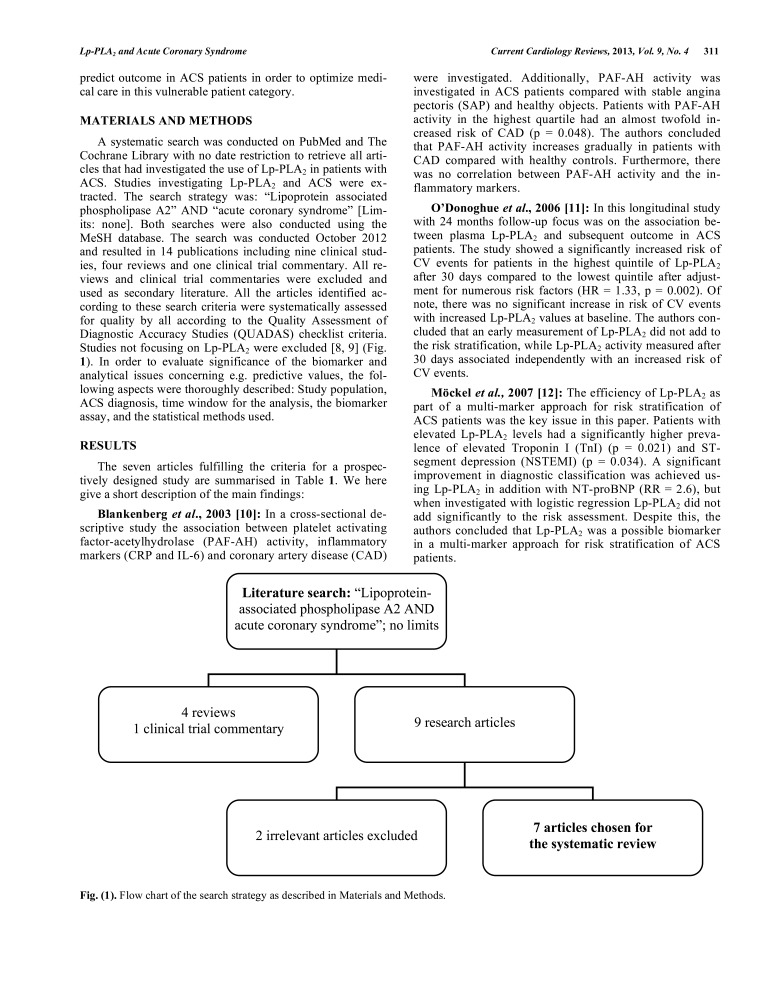
Flow chart of the search strategy as described in Materials and Methods.

A systematic search was conducted on PubMed and The Cochrane Library with no date restriction to retrieve all articles that had investigated the use of Lp-PLA_2_ in patients with ACS. Studies investigating Lp-PLA_2_ and ACS were extracted. The search strategy was: “Lipoprotein associated phospholipase A2” AND “acute coronary syndrome” [Limits: none]. Both searches were also conducted using the MeSH database. The search was conducted October 2012 and resulted in 14 publications including nine clinical studies, four reviews and one clinical trial commentary. All reviews and clinical trial commentaries were excluded and used as secondary literature. All the articles identified according to these search criteria were systematically assessed for quality by all according to the Quality Assessment of Diagnostic Accuracy Studies (QUADAS) checklist criteria. Studies not focusing on Lp-PLA_2_ were excluded [8, 9] (Fig. **1**). In order to evaluate significance of the biomarker and analytical issues concerning e.g. predictive values, the following aspects were thoroughly described: Study population, ACS diagnosis, time window for the analysis, the biomarker assay, and the statistical methods used.

## RESULTS

The seven articles fulfilling the criteria for a prospectively designed study are summarised in Table **1**. We here give a short description of the main findings:

Blankenberg *et al*., 2003 [10]: In a cross-sectional descriptive study the association between platelet activating factor-acetylhydrolase (PAF-AH) activity, inflammatory markers (CRP and IL-6) and coronary artery disease (CAD) were investigated. Additionally, PAF-AH activity was investigated in ACS patients compared with stable angina pectoris (SAP) and healthy objects. Patients with PAF-AH activity in the highest quartile had an almost twofold increased risk of CAD (p = 0.048). The authors concluded that PAF-AH activity increases gradually in patients with CAD compared with healthy controls. Furthermore, there was no correlation between PAF-AH activity and the inflammatory markers.

O’Donoghue *et al*., 2006 [11]: In this longitudinal study with 24 months follow-up focus was on the association between plasma Lp-PLA_2_ and subsequent outcome in ACS patients. The study showed a significantly increased risk of CV events for patients in the highest quintile of Lp-PLA_2_ after 30 days compared to the lowest quintile after adjustment for numerous risk factors (HR = 1.33, p = 0.002). Of note, there was no significant increase in risk of CV events with increased Lp-PLA_2_ values at baseline. The authors concluded that an early measurement of Lp-PLA_2_ did not add to the risk stratification, while Lp-PLA_2_ activity measured after 30 days associated independently with an increased risk of CV events.

Möckel *et al.,* 2007 [12]: The efficiency of Lp-PLA_2_ as part of a multi-marker approach for risk stratification of ACS patients was the key issue in this paper. Patients with elevated Lp-PLA_2_ levels had a significantly higher prevalence of elevated Troponin I (TnI) (p = 0.021) and ST-segment depression (NSTEMI) (p = 0.034). A significant improvement in diagnostic classification was achieved using Lp-PLA_2_ in addition with NT-proBNP (RR = 2.6), but when investigated with logistic regression Lp-PLA_2_ did not add significantly to the risk assessment. Despite this, the authors concluded that Lp-PLA_2_ was a possible biomarker in a multi-marker approach for risk stratification of ACS patients.

Oldgren *et al*., 2007 [13]: This study investigated Lp-PLA_2_ as an independent biomarker of CV events in ACS patients. Furthermore, they evaluated the relationship between Lp-PLA_2_ mass and other known risk markers (e.g. CRP, Troponin T (TnT), and IL-6) in ACS patients compared to healthy subjects. The study showed no significant correlation between Lp-PLA_2_ and CV events after 30 days (p = 0.5) or six months (p = 0.8), and furthermore, they could not show any associations between Lp-PLA_2_ and risk of mortality or future CV events (p = 0.5). The authors concluded that Lp-PLA_2_ correlated with and depended on several other inflammatory risk markers, but were not related to CV events or mortality.

Li *et al*., 2010 [14]: Here, the prognostic significance of plasma Lp-PLA_2_ as a risk factor for ACS was investigated. Additionally, the study also evaluated Lp-PLA_2_ as a risk factor for major adverse cardiac events (MACE) in patients with ACS. The study found elevated Lp-PLA_2_ activity in patients with ACS at baseline (p = 0.027). Elevated Lp-PLA_2_ was associated with higher risk of MACE at follow up (p = 0.033). Patients with a new event had higher Lp-PLA_2_ activity compared to those without (p = 0.04). The authors concluded that Lp-PLA_2_ concentrations contributed to risk discrimination by the strong and independent association with MACE.

Dohi *et al.*, 2011 [15]: The relationship between circulating Lp-PLA_2_ and plaque volume (PV) was investigated in ACS patients following PCI. Circulating Lp-PLA_2_ levels and PV decreased significantly during six months (p < 0.001). The change in PV significantly correlated with the change in Lp-PLA_2 _(r = 0.496, p < 0.001). The authors concluded that circulating Lp-PLA_2_ levels were associated with changes in the coronary plaque volume in ACS patients.

Ryu *et al*., 2012 [16]: This study investigated the use of soluble PLA_2 _and Lp-PLA_2_ levels as biomarkers in risk prediction and association to CV events in patients with ACS. There was no association between the baseline levels of Lp-PLA_2_ and the primary end points death and ACS after 16 weeks, neither for Lp-PLA_2_ mass nor for Lp-PLA_2_ activity. However, baseline sPLA_2_ mass did predict risk of death after multivariable adjustment (p = 0.004). The authors did not conclude whether levels of Lp-PLA_2_ could predict CV events, but the results of the study showed no association.

## DISCUSSION

This is the first systematic review of the use of the new biomarker Lp-PLA_2_ as a predictor of long-term outcome in ACS patients. The main message is that plasma Lp-PLA_2_ concentrations indeed seems to reflect changes in the coronary plaque, but due to the contradictive results in the studies it is far to early to think of using Lp-PLA_2_ as a predictor in this patient category. For example, some studies found that Lp-PLA_2_ concentrations correlated with and depended upon several other inflammatory risk markers [12, 13], while other studies found no correlation between the Lp-PLA_2_ levels and risk of MACE [13, 16]. Interestingly, a majority of the studies found that plasma Lp-PLA_2_ did provide valuable information on, which ACS patients were prone to new events and also could provide valuable information on plaque volume; the latter could be highly interesting in order to monitor treatment efficiency of ACS patients. But despite these promising features, there is no evidence for the use of Lp-PLA_2_ as a predictor in this setting. 

In our systematic literature review we find several issues in the retrieved studies that must be addressed – issues that all ought to be considered before the use of Lp-PLA_2_ as a predictive biomarker can be recommended.

## STUDY POPULATION

The inclusion criteria of the studies were ACS verified by symptoms, ECG or biomarkers. However, different exclusion criteria were used. For example, Li *et al.* excluded patients with diabetes mellitus [14], while other studies excluded patients at young age [11-13]. Importantly, Dohi *et al.* did not describe any exclusion criteria at all [15]. Therefore, it is relevant to consider whether Lp-PLA_2_ is usable for risk stratification in all ACS patients or only in a selected population of ACS patients without certain CV risk factors.

Only three studies included a multinational study population [11, 13, 16]. On the contrary, Dohi *et al.* only included a Japanese population [15], which could be problematic as previous studies have indicated that a genetic mutation (V279F) present in 30% of the Japanese population leads to a reduced risk of CVD/CHD [18]. Furthermore, Li *et al.* included a Chinese population [14], but the prevalence of the mentioned mutation is unknown in this population. It is therefore mandatory to examine differences between nationalities to clarify this issue.

## ACS DEFINITION IN THE STUDY POPULATIONS

When comparing these studies it is relevant to evaluate the definition of ACS and the similarity in the study populations. According to the European Society of Cardiology ACS are defined as a syndrome consisting of acute myocardial infarction with ST-segment elevation (STEMI), acute myocardial infarction without ST-segment elevation (NSTEMI) and unstable angina (UAP) [17]. Several of the studies define ACS according to this [11, 13-16], but Möckel *et al.* included patients with suspected ACS only defined as STEMI and NSTEMI [12], while the ACS definition was not described at all in the study by Blankenberg *et al.* [10]. Except for the latter, the clinical trials thus seem comparable despite the small differences between the studies regarding the European guidelines. Therefore, uniformity in the classification used is mandatory if results from a future multicentre study are to be interpreted.

## TIME WINDOW

Unfortunately, there is very little information regarding the definition of “baseline” in the studies: Is baseline at symptom debut, at the admission time at the hospital, at the blood sampling time, or at the time of diagnosis? Oldgren *et al.* investigated the time delay from symptom debut to blood sampling [13], but did not find any alterations in Lp-PLA_2_ levels. Another time aspect to consider is the length of the follow-up period. Also, when is the best time to measure Lp-PLA_2_ levels? O’Donoghue *et al.* found a difference between Lp-PLA_2_ levels after 7 and 30 days [11]. As the time window is only investigated in two studies, it seems relevant to examine how Lp-PLA_2_ changes over time by conducting a series of more frequent measurements in the follow-up period. A recent study by Ostadal *et al.* [19] demonstrated dynamic alterations in Lp-PLA_2_ levels during the early stages of ACS, which indirectly support the hypothesis of an active role for Lp-PLA_2_ in the pathogenesis of ACS. But without a secure knowledge on alterations over time it will be difficult to interpret findings as earlier described for e.g. cardiac troponins.

## ASSAY PERFORMANCE

A high degree of assay reproducibility is needed in order to provide secure clinical information. Many studies have however found this problematic as mean levels vary considerably from study to study dependent on the assay used. Of note, a recent meta-analysis revealed a significant variation in both mass and activity between assays and studies [20] and in another study the preanalytical stability has been questioned [21]. Altogether, the lack of standardization of assay calibration clearly limits the clinical utility of this biomarker, and effort must be put into constructing a more solid assay with a standardized setup.

## THE CLINICAL USE OF Lp-PLA_2_

To evaluate the clinical use of Lp-PLA_2_ it is important to consider the usability of Lp-PLA_2_ as a risk marker of new CV events in ACS patients, the optimal time window and whether it is independent or should be part of a multi-marker approach. Most studies used a direct approach to show a positive correlation between elevated Lp-PLA_2_ plasma concentrations and CV events [10, 12-14, 16], and Dohi *et al.* also evaluated plaque volume, an indirect risk factor of developing ACS [15]. O’Donoghue *et al.* showed that Lp-PLA_2_ was not an effective marker for risk stratification of ACS patients in the acute phase [11]. However, after 30 days Lp-PLA_2_ seemed to be a potential independent marker for CV events. Three of the studies described that independent biomarkers such as TnI, CRP, IL-6 and Lp-PLA_2_ reflected different pathways in the pathophysiology of ACS [10, 12, 13]. It therefore seems relevant to investigate whether Lp-PLA_2_ is usable as an independent biomarker or if a multi-marker approach will give the most applicable results. But before this is possible, the aforementioned issues need to be dealt with.

## STATINS

It is known that Lp-PLA2 activity and mass both are strongly associated with various lipid markers due to a physical binding to LDL cholesterol. Studies have evaluated whether statins or direct Lp-PLA_2_ inhibitors lowering the level of serum Lp-PLA_2_ could be used to minimize plaque volume and thereby the risk of a CV event. At present the direct Lp-PLA_2_ inhibitor Darapladib (a selective blocker of the active serine residue in Lp-PLA_2 _which decreases the activity) is investigated in a phase III study that examines the effect of Darapladib on different cardiovascular outcomes [4, 22-24]. This type of study will be able to tell us whether Lp-PLA_2_ also can be used for monitoring the treatment efficiency.

## CONCLUSIONS

Despite promising results in some of the retrieved studies and a feeling of excitement over this “new-kid-on-the-block” biomarker, there are inconsistencies in the findings regarding a potential use of Lp-PLA_2 _in this setting. Also, there is a huge need for focused studies on the issues lined out above, primarily genetic variations, time-window impact, patients with and without CV risk factors (diabetic patients?), and the assay performance. Nevertheless, if Lp-PLA_2_ really does reflect threatening plaque rupture it still offers new insight in the pathophysiological development of ACS. This will however mainly be of interest in a research setting and not in a clinical setting. For the time being this marker must therefore remain an interesting candidate in this relation.

## Figures and Tables

**Table 1.** Articles retrieved in the systematic literature search.

Study	Type	Year	Place	Study population	Follow-up	Marker	Inclusion criteria	Exclusion criteria	Adjustments	Primary endpoint
Ryu *et al.*	RCT	2012	International	MIRACL trial: 2587	16 weeks	Lp-PLA2 mass and activity	UAP or non-Q-wave acute MI	Elevated serum cholesterol, previous MI, PCI, treatment with lipid-lowering drugs	Age, sex, entry event, treatment group, baseline TnT, smoking, systolic blood pressure, hypertension, DM, previous MI, BMI, LDL-cholesterol, and CRP	CV events: death, nonfatal acute MI, and cardiac arrest with resuscitation or re-hospitalization for UAP
Dohi *et al.*	RCT	2011	Japan	40 patients	6 months	Lp-PLA2 activity and PV	ACS, coronary plaque of a non-PCI site in a culprit vessel	-	-	-
Li *et al.*	Case-control study	2011	China	152 patients and 142 controls	6 months	Lp-PLA2 activity	ACS diagnosed by ECG and troponins	Infection, systemic immune disease, liver cirrhosis, renal dysfunction, DM, malicious tumor, and cerebrovascular disease	Age and sex	MACE: CV death, non-fatal MI, and target vessel re-vascularization
Oldgren *et al.*	Case-control study	2007	International	FRISC-II: 1362 patients GUSTO IV: 904 patients SWISH study: 435 controls	FRISC-II: 6 months and 2 years GUSTO IV: 30 days	Lp-PLA2 mass	FRISC II: Symptoms of ischemia within 48 h before the start of dalteparin or unfractionated heparin treatment GUSTO IV: Over 21 years with chest pain lasting > 5 min and either positive troponins or NSTEMI	FRISC-II: raised risk of bleeding episodes, server cardiac disease, renal or hepatic insufficiency, patients with previous open-heart surgery etc. GUSTO-IV: MI precipitated by other disorders than atherosclerotic CAD, persistent STEMI, newly or planned PCI or CABG, history of stroke etc.	-	CV events: death, MI and mortality
MÖckel *et al.*	Prospective study	2007	Germany	429 patients	42 days	Lp-PLA2 mass	Suspicion of ACS diagnosed by the attending physician	Severe anaemia, age of less than 18 years etc.	-	MACE: Death, non-fatal MI, UAP requiring admission, PCI, CABG, etc.
O’Donoghue *et al.*	Cohort study	2006	International	4162 patients	7 and 30 days	Lp-PLA2 mass and activity	MI, age more than 18 years, low total cholesterol levels	Coexisting condition that shortened expected survival, statin therapy (80 mg), PCI (6 month), prolonged QT-interval, hepatic disease etc.	Cardiac risk factors, treatment, LDL and CRP	MACE: Death, MI, UAP, re-vascularization, or stroke
Blankenberg *et al.*	Cross-sectional study	2003	Germany	973 patients	-	PAF-AH activity	Stenosis diameter >30% in at least one major coronary artery.	Significant concomitant disease (cardiomyopathy, malignant disease and febrile condition)	Clinical factors, metabolic factors, sex, age, BMI, smoking, hypertension, lipid parameters	CAD: SAP and ACS

RCT, randomized clinical trial; Lp-PLA2, lipoprotein phospholipase A2; UAP, unstable angina pectoris; MI, Myocardial infarct; PCI, percutane coronary intervention; TnT, Troponin T; DM, diabetes mellitus; BMI, body mass index; LDL, low density lipoprotein; CRP, C-reactive protein; CV, cardio vascular; PV, Plaque volume; ACS, acute coronary syndrome; ECG, electrocardiography; TnI, Troponin I; NSTEMI, ST-segment depression myocardial infarct; MACE, major adverse cardiac events; STEMI, ST-segment elevation myocardial infarct; CABG, coronary artery bypass graft; PAF-AH, Platelet activating factor-acetylhydrolase; CAD, coronary artery disease; SAP, stabile angina pectoris.
